# Association of ATRX with pericentric heterochromatin and the Y chromosome of neonatal mouse spermatogonia

**DOI:** 10.1186/1471-2199-9-29

**Published:** 2008-03-13

**Authors:** Claudia Baumann, Anja Schmidtmann, Kathrin Muegge, Rabindranath De La Fuente

**Affiliations:** 1 Female Germ Cell Biology Group, Department of Clinical Studies, Center for Animal Transgenesis and Germ Cell Research, School of Veterinary Medicine, University of Pennsylvania, New Bolton Center, 382 West Street Road, Kennett Square, PA 19348, USA; 2 Laboratory of Cancer Prevention, SAIC-Basic Research Program, National Cancer Institute, Frederick, MD 21701, USA

## Abstract

**Background:**

Establishment of chromosomal cytosine methylation and histone methylation patterns are critical epigenetic modifications required for heterochromatin formation in the mammalian genome. However, the nature of the primary signal(s) targeting DNA methylation at specific genomic regions is not clear. Notably, whether histone methylation and/or chromatin remodeling proteins play a role in the establishment of DNA methylation during gametogenesis is not known. The chromosomes of mouse neonatal spermatogonia display a unique pattern of 5-methyl cytosine staining whereby centromeric heterochromatin is hypo-methylated whereas chromatids are strongly methylated. Thus, in order to gain some insight into the relationship between global DNA and histone methylation in the germ line we have used neonatal spermatogonia as a model to determine whether these unique chromosomal DNA methylation patterns are also reflected by concomitant changes in histone methylation.

**Results:**

Our results demonstrate that histone H3 tri-methylated at lysine 9 (H3K9_me3_), a hallmark of constitutive heterochromatin, as well as the chromatin remodeling protein ATRX remained associated with pericentric heterochromatin regions in spite of their extensive hypo-methylation. This suggests that in neonatal spermatogonia, chromosomal 5-methyl cytosine patterns are regulated independently of changes in histone methylation, potentially reflecting a crucial mechanism to maintain pericentric heterochromatin silencing. Furthermore, chromatin immunoprecipitation and fluorescence in situ hybridization, revealed that ATRX as well as H3K9_me3 _associate with Y chromosome-specific DNA sequences and decorate both arms of the Y chromosome, suggesting a possible role in heterochromatinization and the predominant transcriptional quiescence of this chromosome during spermatogenesis.

**Conclusion:**

These results are consistent with a role for histone modifications and chromatin remodeling proteins such as ATRX in maintaining transcriptional repression at constitutive heterochromatin domains in the absence of 5-methyl cytosine and provide evidence suggesting that the establishment and/or maintenance of repressive histone and chromatin modifications at pericentric heterochromatin following genome-wide epigenetic reprogramming in the germ line may precede the establishment of chromosomal 5-methyl cytosine patterns as a genomic silencing strategy in neonatal spermatogonia.

## Background

In the mammalian germ line, parental-specific genomic imprints are erased during each generation in order to allow for their subsequent re-establishment during gametogenesis [1]. This process of epigenetic reprogramming is initiated during mouse embryonic development at around day 10.5 post coitum (p.c.), a time when primordial germ cells migrate to the genital ridge [2-4]. Re-establishment of sex-specific epigenetic modifications in the male germ line begins on days 13–17 p.c. and continues during the first days of postnatal development in mitotically dividing neonatal spermatogonia [5-9]. Importantly, in contrast with the chromosomes of somatic cells, which exhibit a characteristic global DNA methylation status (as determined by 5-methyl cytosine; 5-mC staining) with weakly methylated chromatids and strongly methylated centromeric regions, the chromosomes of neonatal spermatogonia exhibit a unique DNA methylation pattern [10]. For example, centromeric heterochromatin in spermatogonial cells is hypo-methylated, whereas euchromatin regions convert from a demethylated to a strongly methylated status between days 16 to 17 p.c. in the mouse germ line [10]. Therefore, immediately after birth, the chromatids of germ cell chromosomes appear hyper-methylated whereas centromeric domains are globally demethylated [11,12].

In lower organisms such as *Neurospora crassa *and *Arabidopsis thaliana *the patterns of genomic DNA methylation are directly influenced by histone methylation [13-15]. Evidence obtained from the *Arabidopsis *model indicates that establishment of DNA methylation patterns is essential for the subsequent di-methylation of histone H3 (H3K9_me2_) [16]. In contrast, targeted deletion of the two isoforms of *Suv39h*, a histone methyltransferase specifically involved in tri-methylation of histone H3 at lysine 9 (H3K9_me3_), results in altered DNA methylation of tandem repeats at pericentric heterochromatin in murine embryonic stem cells suggesting that in mammals histone tri-methylation (H3K9_me3_) might be required for the establishment and/or maintenance of DNA methylation [15,17,18]. Although some aspects of the functional interaction between histone methylation and DNA methylation have, to some extent, been evolutionarily conserved in mammals, the precise relationship between H3K9_me3 _and DNA methylation is not fully understood.

Tri-methylation of histone H3 on lysine 9 (H3K9_me3_) is a hallmark of pericentric heterochromatin [19]. Importantly, H3K9_me3 _provides a docking site for additional chromatin binding proteins such as heterochromatin protein 1 (HP1) in an essential step for heterochromatin formation and the maintenance of a transcriptionally repressive environment [20-23]. In contrast, di-methylation of histone H3 at lysine 4 (H3K4_me2_) is associated with transcriptionally permissive euchromatin regions in the genome [24,25].

Notably, recent evidence indicates that helicases of the SWI/SNF2 family of chromatin remodeling proteins such as ATRX (alpha thalassemia/mental retardation syndrome X-linked) and the lymphoid specific helicase (LSH), also known as helicase lymphoid specific (Hells) bind to pericentric heterochromatin regions in mouse somatic cell lines where they also play an essential role in DNA methylation [26-31]. However, whether these chromatin remodeling proteins play a role in heterochromatin formation in the male germ line is not known.

To gain insight into the relationship between global DNA methylation and histone methylation in the spermatogonial cell genome, we determined whether the unique DNA methylation patterns observed in the chromosomes of neonatal male germ cells are also reflected by similar changes in histone methylation and whether the extensive de-methylation of centromeric domains interferes with the association of heterochromatin binding proteins in mitotic germ cell chromosomes. Our results indicate that both H3K9_me3 _as well as ATRX remained associated with pericentric heterochromatin in spermatogonial cell autosomes regardless of the chromosomal DNA methylation status. Moreover, we found, that the high levels of global DNA methylation (5-mC) in germ cell chromatids are not necessarily coupled with changes in histone methylation. These results suggest that during early postnatal development of the male mitotic germ cell, global or chromosomal DNA methylation patterns are regulated independently of changes in histone methylation. Additionally, we found that ATRX and H3K9_me3 _mark the entire Y chromosome in neonatal spermatogonia. Our results are discussed within the context of the ontogeny of ATRX and its dynamic interactions with chromatin remodeling and transcriptional regulatory complexes during spermatogenesis as well as the potential implications for heterochromatinization of the Y chromosome.

## Results

### Characterization of histone H3 methylation patterns and ATRX localization in the chromosomes of neonatal spermatogonia

**Figure 1:**
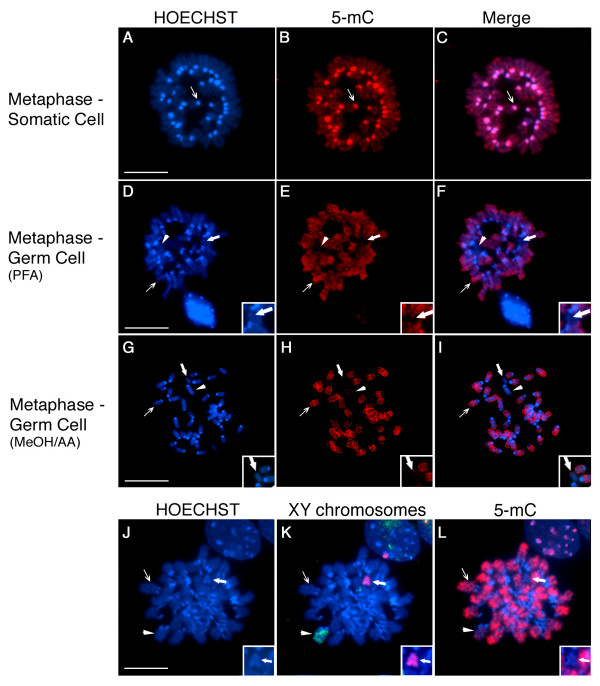
**Comparison of Global DNA methylation patterns in the chromosomes of mouse embryonic fibroblasts and neonatal spermatogonia**. **A-C) **Fluorescent micrographs of metaphase chromosomes from primary mouse embryonic fibroblasts showing highly methylated autosomal centromeric domains (thin arrow) as determined by 5-methyl cytosine (5-mC) staining (red). **D-F) **Chromosomes in neonatal spermatogonia lack global DNA methylation at pericentric heterochromatin, while 5-mC (red) decorates entire chromatids in the majority of autosomes (thin arrow). Two chromosomes on each metaphase spread consistently exhibit lack of 5-mC staining (bold arrow and arrowhead). These patterns are reproducible after using two standard chromosome fixation protocols using either paraformaldehyde (PFA) or methanol/acetic acid (MeOH/AA) as shown in **(G-I)**. **J-L) **Fluorescent in situ hybridization (FISH) and subsequent determination of DNA methylation patterns (5-mC; red) confirms that the two hypo-methylated chromosomes observed on each spread correspond to the X chromosome (green; arrow head) and the Y chromosome (red; Inset and bold arrow in K-L). Note the lack of 5-mC staining on the Y chromosome and the low levels of global methylation on the X chromosome compared to autosomes (thin arrows) in germ cell metaphases. DNA was counterstained with HOECHST 33258 (blue). Scale bars = 10 μm.

The primary signals responsible for establishing and maintaining global DNA methylation and histone methylation patterns at the level of distinct chromosomal domains in the mammalian genome are not known. However, recent evidence obtained from the analysis of somatic cells and embryonic stem cells in mammals indicates the existence of a functional interaction between tri-methylation of lysine 9 on histone H3 (H3K9_me3_) and DNA methylation at pericentric heterochromatin domains [15,17,18,32]. In somatic cells, pericentric heterochromatin domains exhibit high levels of DNA methylation as determined by 5-mC staining [33] and Figure 1A–C (arrow), whereas chromatids of metaphase chromosomes exhibit only a faint banding pattern. In contrast, except for the presence of two chromosomes that appear globally demethylated (arrowhead and bold arrow; Figure 1D–F), the majority of chromosomes in neonatal spermatogonia exhibit a unique pattern of 5-mC staining in which chromatids appear highly methylated while centromeric domains lack 5-mC staining [10-12] and (thin arrow in Figure 1D–F). These patterns are reproducible after cross-linking of chromosomal proteins with paraformaldehyde (PFA; Figure 1D–F), which is essential for subsequent detection of histone post-translational modifications, or after treatment of cells with a hypotonic solution and chromosome fixation with methanol/acetic acid (MeOH/AA), which improves chromosome spreading on the metaphase plate (Figure 1G–I) and thus facilitates the subsequent identification of individual chromosomes after fluorescence in situ hybridization (FISH). Simultaneous analysis of 5-mC patterns and FISH with an X chromosome specific probe (green) or a Y chromosome specific probe (red; Figure 1J–L) demonstrates that compared with the rest of the autosomes, the X chromosome (arrowhead) is hypo-methylated whereas the Y chromosome (bold arrow) exhibits a global demethylation pattern in neonatal spermatogonia (Figure 1K–L; Inset).

**Figure 2:**
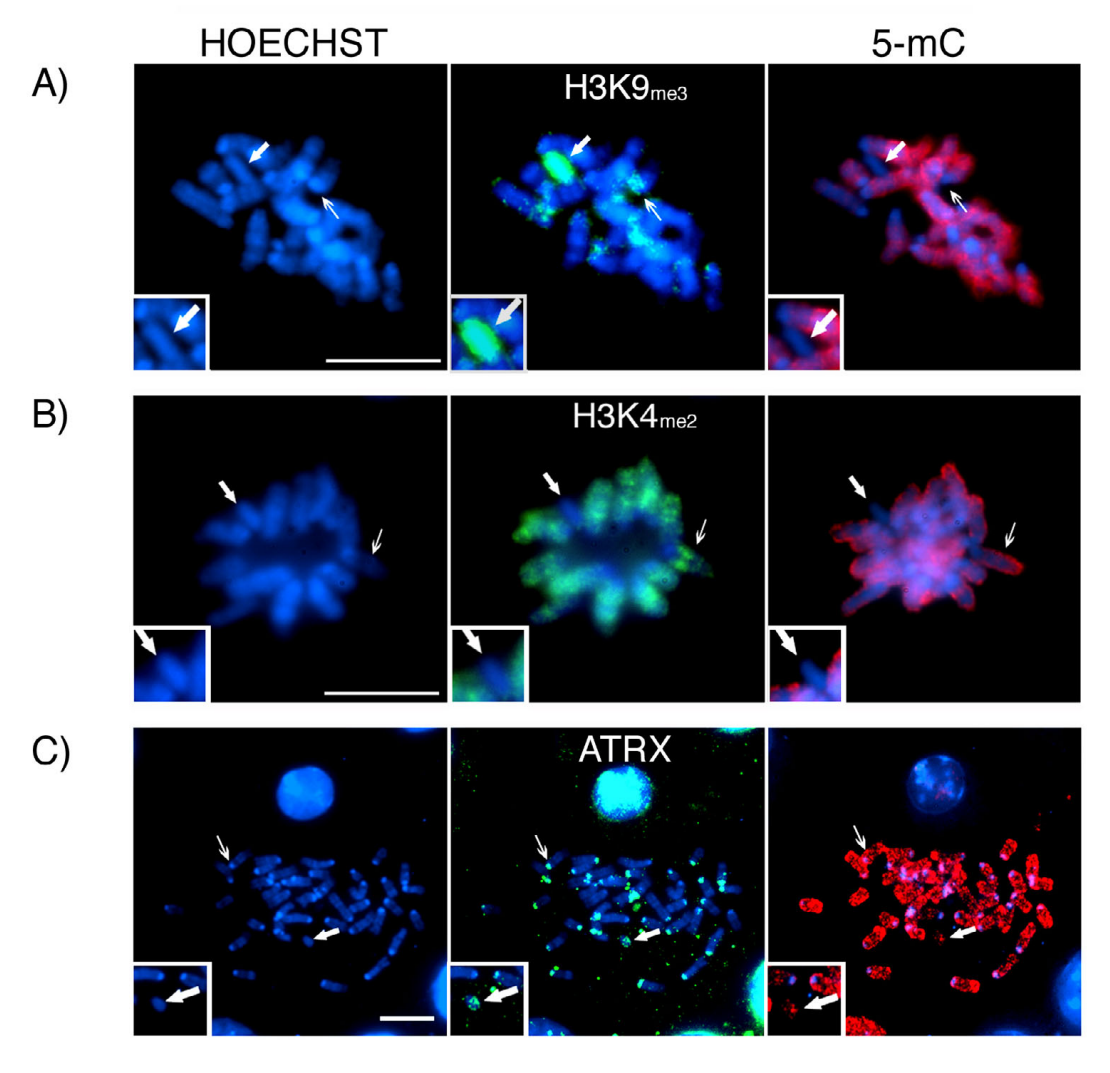
**Chromosomal localization of ATRX and histone H3 tri-methylation on lysine 9 (H3K9_me3_) in neonatal spermatogonia**. **A) **Analysis of histone methylation patterns revealed that in spite of the absence of global DNA methylation (red) at pericentric heterochromatin (thin arrow), H3K9_me3 _(green) remained associated with centromeric domains in the chromosomes of neonatal spermatogonia. Importantly, H3K9_me3 _is also preferentially associated with one of the unmethylated chromosomes on each spread analyzed (bold arrow). **B) **In contrast, H3K4_me2 _(green) remains associated with entire chromatids in the majority of chromosomes (thin arrow). However, one of the unmethylated chromosomes was consistently found to be completely devoid of H3K4_me2 _(bold arrow). **C) **Notably, the chromatin remodeling protein ATRX (green) remains associated with pericentric heterochromatin and consistently marks a single unmethylated chromosome on each metaphase spread analyzed. Scale bar = 10 μm.

To determine whether lack of DNA methylation at pericentric heterochromatin domains in neonatal spermatogonia alters histone methylation patterns, we assessed the distribution of H3K9_me3 _as well as H3K4_me2 _in surface spread metaphase chromosomes obtained from male pups on day 2 of postnatal development. The characteristic patterns of 5-mC staining in neonatal spermatogonia are a reliable marker to distinguish germ cell-derived chromosomes from those of somatic cell origin (Figure 2A–C). In spite of their extensive demethylation in the germ cell lineage, pericentric heterochromatin domains exhibit a precise staining for H3K9_me3 _in all autosomes (Figure 2A; thin arrow). Interestingly, one chromosome on each metaphase spread analyzed consistently presented a preferential accumulation of H3K9_me3 _throughout the length of both chromatids (Figure 2A; Inset). This chromosome also appeared globally demethylated as determined by the patterns of 5-mC staining (Figure 2A; Inset and bold arrow). In contrast, the patterns of H3K4_me2_, a marker of transcriptionally permissive euchromatic regions, showed a reciprocal association with germ cell chromosomes. In spite of their extensive 5-mC staining, H3K4_me2_remained faithfully associated with the chromatids of neonatal spermatogonia (Figure 2B; thin arrow) but failed to associate with pericentric heterochromatin domains. Notably, in every metaphase spread analyzed, H3K4_me2 _was entirely absent from one of the chromosomes showing global demethylation (Figure 2B; Inset and bold arrow).

In addition to histone methylation, helicases of the SWI/SNF2 protein family reviewed in [34] such as ATRX might play a critical role in heterochromatin formation as well as in the establishment and/or maintenance of specific methylation patterns [27]. Thus we determined whether the methylation patterns present in neonatal spermatogonia affect the recruitment of ATRX to pericentric heterochromatin. Similar to H3K9_me3_, ATRX remains faithfully associated with pericentric heterochromatin in the autosomes of neonatal germ cells (Figure 2C; thin arrow). Notably, one of the sex chromosomes, which exhibit global demethylation, consistently displayed a bright ATRX signal in all metaphase spreads analyzed (Figure 2C; Inset and bold arrow). Taken together, these results suggest that in spite of extensive DNA demethylation at pericentric heterochromatin domains in the chromosomes of neonatal spermatogonia, repressive histone modifications such as H3K9_me3 _are already established and that chromatin-binding proteins essential to maintain a transcriptionally repressive environment, such as ATRX are efficiently recruited to pericentric heterochromatin.

### ATRX and H3K9_me3 _mark the Y chromosome in neonatal spermatogonia

**Figure 3:**
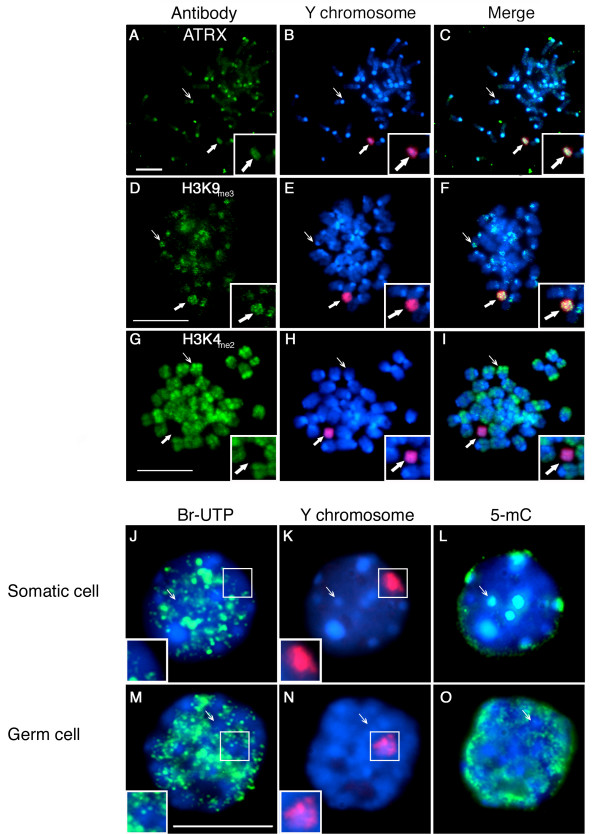
**Repressive histone and chromatin modifications in the absence of global DNA methylation in the Y chromosome of neonatal spermatogonia**. **A-C) **Immuno-FISH analysis of neonatal spermatogonia revealed that the ATRX protein (green) as well as H3K9_me3 _(green; **D-F**) consistently associate with pericentric heterochromatin domains in autosomes (thin arrows). In addition to their centromeric localization, ATRX as well as H3K9_me3 _preferentially associate with both arms of the Y chromosome (red; bold arrows). **G-I) **In contrast, a transcriptionally permissive histone modification such as H3K4_me2 _(green) exhibited an inverted chromosomal distribution whereby constitutive heterochromatin (i.e. pericentric heterochromatin and the Y chromosome; red) are completely devoid of H3K4_me2_. The Y chromosome in neonatal spermatogonia occupies a transcriptionally quiescent nuclear domain during interphase. Transcription run-on assays after incorporation of Br-UTP (green) in permeabilized interphase nuclei of somatic **(J-L) **and neonatal spermatogonia **(M-O) **revealed that global transcriptional activity is undetectable in the nuclear domain occupied by the Y chromosome (red; see inset) in both somatic cell and germ cell nuclei. Neonatal spermatogonial cell nuclei were identified by their unique global DNA methylation patterns (5-mC; green). Scale bar = 10 μm.

To determine which of the sex chromosomes showing global DNA hypo-methylation is preferentially labeled by ATRX and H3K9_me3 _we analyzed metaphase spreads using immunofluorescence followed by FISH in order to establish the position of the Y chromosome. Consistent with our previous experiments, ATRX binds to pericentric heterochromatin domains in the chromosomes of neonatal spermatogonia (Figure 3A; thin arrow). Importantly, one chromosome on each metaphase spread exhibits prominent ATRX labeling encompassing the entire length of both chromatid arms (Figure 3A; Inset and bold arrow). Subsequent analysis with a Y chromosome-specific probe on the same metaphase spread revealed that the prominent ATRX staining corresponds to the chromatids of the Y chromosome in all spreads analyzed (N = 108), (Figure 3A–C; Inset and bold arrow). Moreover, analysis of histone methylation patterns revealed that while the Y chromosome also exhibits prominent staining with H3K9_me3_, (Figure 3D–F; Inset), no staining for H3K4_me2 _was detectable in the chromatids of the Y chromosome in any of the metaphase spreads analyzed (N = 98) Figure 3G–I (Inset and bold arrow). These results indicate that in neonatal spermatogonia, pericentric heterochromatin domains as well as the Y chromosome preferentially associate with transcriptionally repressive histone and chromatin modifications. In addition, these nuclear domains also lack any association with transcriptionally permissive histone modifications such as H3K4_me2._

At the pachytene stage of meiosis the X and Y chromosomes reside in a transcriptionally inactive nuclear domain at the sex body [35,36]. Importantly, global transcriptional silencing associated with the Y chromosome has also been recently observed in types A and B spermatogonia obtained from adult mice [37]. However, the transcriptional status of the nuclear domain occupied by the Y chromosome during interphase in neonatal spermatogonia has not been determined. Analysis of global transcriptional activity after Br-UTP incorporation into nascent transcripts (Figure 3J; green) in somatic testicular cells revealed several heterochromatic domains with no detectable transcription (Figure 3J; Inset). Moreover, the nuclear domain occupied by the Y chromosome (Figure 3K; red) during interphase remains transcriptionally quiescent (Figure 3J; Inset). Similar results were observed in the nucleus of neonatal spermatogonia in which the Y chromosome (red) also occupies a transcriptionally silent nuclear domain as determined by the lack of Br-UTP incorporation (Figure 3M–N; Inset). This suggests that the Y chromosome in neonatal spermatogonia is subject to unique chromatin modifications that allow for the accumulation of heterochromatin marks associated with transcriptional repression such as ATRX and H3K9_me3. _These marks accumulate in spite of a global DNA hypo-methylation status comprising the entire chromosome suggesting an independent regulation of histone and DNA methylation in neonatal germ cells.

### Association of ATRX and H3K9_me3 _with repetitive DNA sequences on the Y chromosome

**Figure 4:**
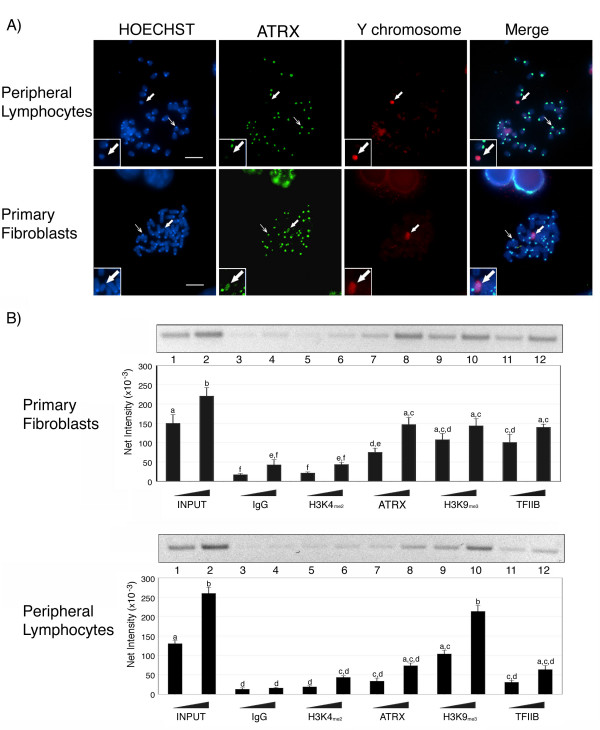
**Specific association of ATRX and H3K9_me3 _with lack of H3K4_me2 _at repetitive DNA sequences on the Y chromosome**. **A) **Metaphase spreads of peripheral lymphocytes (top panel) and primary fibroblasts (lower panel). Thin arrows indicate the characteristic association of ATRX (green) at pericentric heterochromatin in the autosomes of both cell lineages. ATRX consistently labels the Y chromosome (red) in mouse embryonic fibroblasts (bold arrow) but is undetectable in the Y chromosome of peripheral lymphocytes. Scale bars = 10 μm. **B) **Chromatin immunoprecipitation (ChIP) analysis of histone modifications associated with a Y-chromosome specific repetitive sequence. ChIP assays were performed on male embryonic fibroblasts (upper panel) and peripheral lymphocytes (lower panel) using antibodies directed against ATRX, H3K4_me2 _and H3K9_me3_. Representative gel images are depicted above the corresponding graphs. A rabbit IgG (lanes 3 and 4) and an anti-TFIIB antibody (lanes 11 and 12) served as negative and positive control, respectively. In primary fibroblasts, the specific association of H3K9_me3 _(lanes 9 and 10) and ATRX (lanes 7 and 8) with repetitive sequences on the Y chromosome results in a significant enrichment of co-precipitated DNA fragments corresponding to the Y chromosome-specific repeat (Y666) compared to a non-specific IgG negative control (lanes 3 and 4) and the H3K4_me2 _antibody (lanes 5 and 6). However, although H3K9_me3 _is also enriched at these sequences in peripheral lymphocytes (lower panel), ATRX associations are not significantly different from samples precipitated with the negative control (IgG) or H3K4_me2_. PCR amplification was conducted using increasing amounts of precipitated template (input) to ensure that the PCR reaction was within the linear range. Error bars represent the SEM of three independent experiments and different superscripts indicate significant differences (p < 0.05).

To determine whether the localization of ATRX with the Y chromosome is restricted to the germ line, we set out to analyze the epigenetic composition of the Y chromosome in metaphase spreads obtained from primary cultures of two different somatic tissues. Analysis of peripheral lymphocytes indicates that ATRX is localized to pericentric heterochromatin in the majority of chromosomes (thin arrow in Figure 4A; upper panel). However, in these cells, ATRX staining was undetectable on the Y chromosome (Figure 4A; upper panel; Inset and bold arrow). In contrast, ATRX was localized at pericentric heterochromatin and prominently to the Y chromosome in primary embryonic fibroblasts (Figure 4A; lower panel; Inset and bold arrow). Notably, the global demethylation observed on the Y chromosome of embryonic fibroblasts also contrasts with that of the Y chromosome in peripheral lymphocytes in which it remains methylated as indicated by 5-mC staining (see Additional file 1). These results suggest that the localization of ATRX to the Y chromosome is not restricted to the germ line and that there might be cell type or tissue-specific differences in the epigenetic composition of the Y chromosome in the soma.

Next, using chromatin immunoprecipitation (ChIP) followed by PCR analysis, we determined whether ATRX and H3K9_me3 _exhibit a specific association with pericentric repetitive DNA sequences on the murine Y chromosome. Enzymatically digested chromatin derived from embryonic fibroblast nuclei and peripheral lymphocytes was immunoprecipitated using ATRX or H3K9_me3 _antibodies. To validate our system we first examined the precipitates for the presence of rDNA promoter sequences that had been previously reported to interact with the ATRX protein [27]. Using two different antibodies against the ATRX protein we confirmed a specific association of ATRX with rDNA (Data not shown). After immunoprecipitation with anti-ATRX antibodies, pericentric repeat sequences specific to the Y chromosome were significantly enriched in primary fibroblasts (Figure 4B, upper panel; lanes 7 and 8; p < 0.05), whereas a negative control precipitation using pre-immune IgG resulted only in a background level of amplification. Similarly, Y-specific repeat sequences were also enriched after immunoprecipitation of chromatin with an anti-H3K9_me3 _antibody (Figure 4B, upper panel; lanes 9 and 10). In contrast, and consistent with our immuno-FISH results, ChIP analysis using the H3K4_me2 _antibody (lanes 5 and 6) failed to enrich for Y-chromosome specific DNA sequences and showed only basal levels, undistinguishable from those observed in the negative IgG control group (compare lanes 3–4 to 5–6).

Immunoprecipitation of peripheral lymphocyte chromatin with anti-H3K9_me3 _antibody resulted in a significant enrichment (p < 0.05) of Y chromosome-specific repeat sequences (Figure 4B, lower panel; lanes 9 and 10). However, ChIP analysis using anti-ATRX (Figure 4B, lower panel; lanes 7 and 8) and anti-H3K4_me2 _antibodies (lower panel; lanes 5 and 6) failed to enrich for Y-chromosome specific DNA sequences and showed no significant differences with the negative control (IgG) group (lower panel, lanes 3–4). These results are consistent with our immuno-FISH data and indicate that cell type-specific associations of ATRX with the Y chromosome can be observed not only on a chromosome-wide basis but also at the molecular level.

### Localization of ATRX and H3K9_me3 _to centromeric heterochromatin and the Y chromosome in neonatal spermatogonia of LSH deficient mice

**Figure 5:**
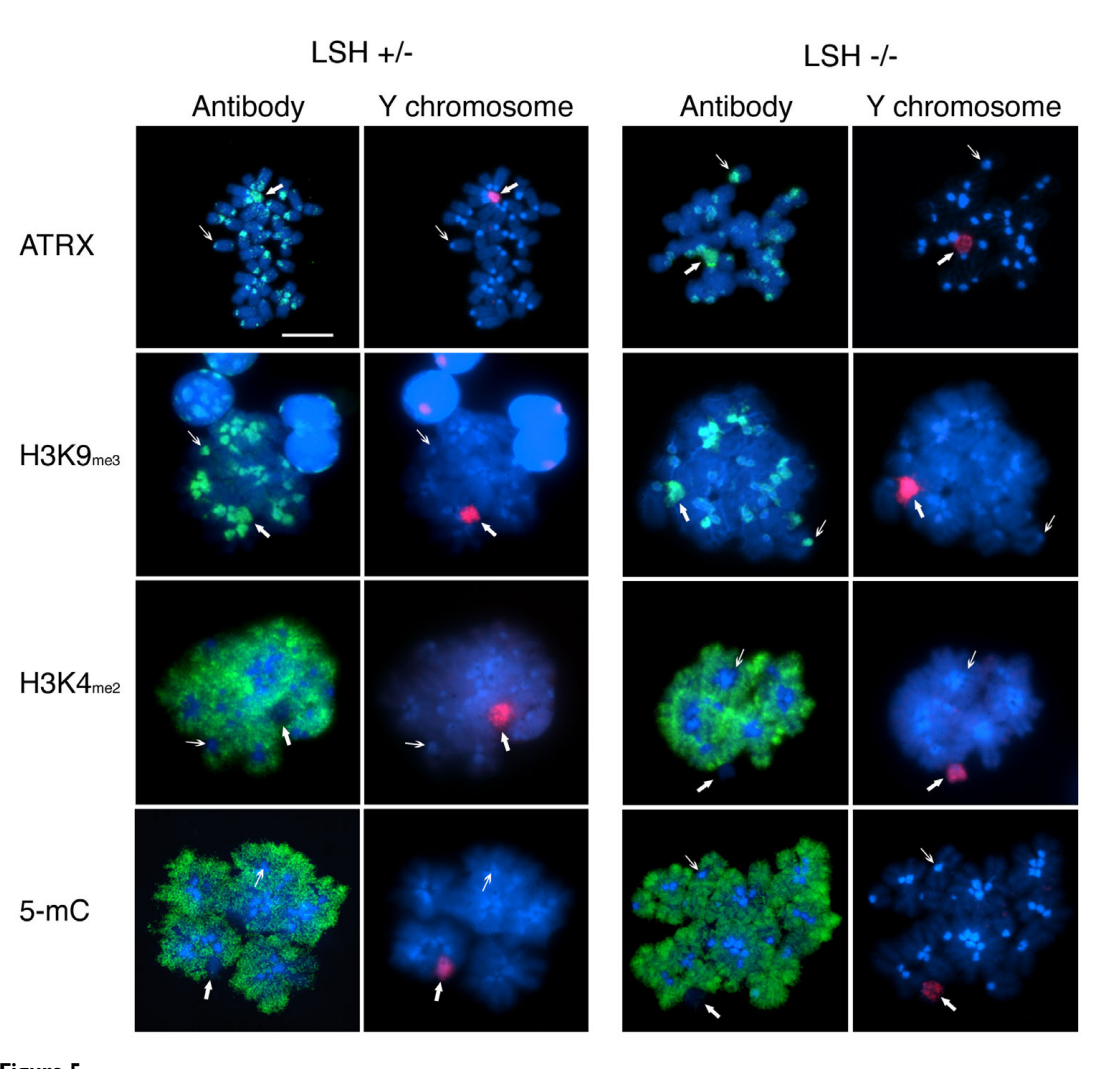
**Binding of ATRX to centromeric heterochromatin and the Y chromosome in neonatal spermatogonia from LSH ^-/- ^mice**. Neonatal spermatogonia were obtained from heterozygous controls and LSH deficient mice and analyzed by Immuno-FISH. The sub-chromosomal localization of ATRX, H3K9_me3_, H3K4_me2 _and 5-mC were correlated with the position of the Y chromosome (red). The patterns of ATRX and H3K9_me3 _localization to centromeric domains (thin arrows) and the Y chromosome (bold arrow) in cells lacking LSH protein were undistinguishable compared to the patterns observed in heterozygous littermate controls. Neonatal spermatogonial cell nuclei were identified by their unique global DNA methylation patterns (5-mC; green) on the same metaphase spread. Scale bar = 10 μm.

In the male germ cell lineage, the patterns of global DNA methylation are erased during fetal development and progressively reestablished shortly after birth [3,4,7,38]. Notably, the lymphoid specific helicase (LSH) has recently been implicated in the establishment of *de novo *DNA methylation patterns in murine embryonic stem cells [39]. Furthermore, lack of LSH function results in abnormal DNA methylation at repetitive elements including major satellite sequences at pericentric heterochromatin as well as single copy genes throughout the genome [28]. Thus we set out to determine whether the unique patterns of global 5-mC staining present in the chromosomes of neonatal spermatogonia might be affected by the lack of LSH function. Consistent with our previous experiments, ATRX and H3K9_me3 _co-localized at pericentric heterochromatin regions in the autosomes as well as the Y chromosome of control heterozygous spermatogonial cells maintained in culture to a stage equivalent to day 1 of postnatal development (Figure 5). Moreover, H3K4_me2 _and 5-mC staining were detected exclusively in the chromatid arms of most chromosomes, but were excluded from both centromeric domains as well as the Y chromosome. Similar results were obtained after analysis of LSH (-/-) spermatogonia (Figure 5). This suggests that neither the patterns of H3K9_me3 _nor ATRX localization at pericentric heterochromatin and the Y chromosome were affected by the lack of LSH function in neonatal spermatogonia. Notably, although the lack of LSH function results in abnormal DNA methylation in somatic cells [28], our results indicate that the patterns of intense 5-mC staining observed throughout the chromatids of neonatal spermatogonia were not affected by the absence of LSH protein.

### Sexual dimorphism in the patterns of ATRX nuclear compartmentalization during meiosis

**Figure 6:**
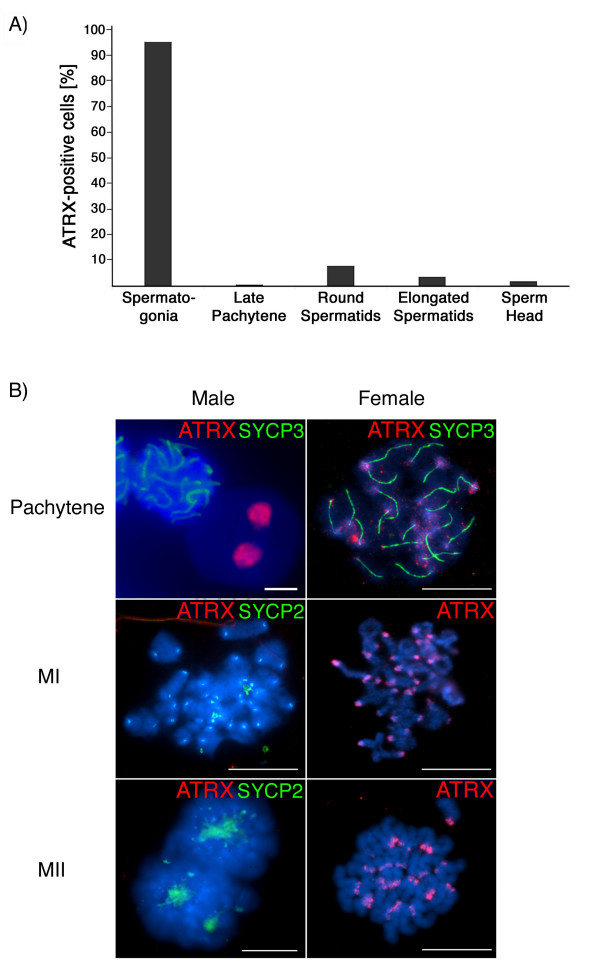
**Sexual dimorphism in the patterns of ATRX nuclear localization during mouse meiosis**. **A) **Proportion of cells that exhibit ATRX nuclear staining in surface spread adult spermatogenic cells. Although ATRX is found at centromeric domains in the majority of adult spermatogonial cells, this protein is notably absent from the nucleus of pachytene spermatocytes, expressed in a small proportion (< 10%) or round spermatids and only occasionally detectable in the nucleus of elongated spermatids and condensed sperm heads. Data are presented as the mean percentage of cells with nuclear ATRX staining from 3 independent experiments. **B) **Dynamics of ATRX nuclear localization during mouse spermatogenesis. ATRX (red) binds pericentric heterochromatin domains in pachytene stage oocytes showing fully synapsed chromosomes stained with the synaptonemal complex protein SYCP3 (green). In contrast ATRX is notably absent from pachytene stage spermatocytes and only detectable at the chromocenter of stage 8–9 round spermatids. ATRX remains associated with the centromeres of meiotic chromosomes in metaphase I (MI) and metaphase II (MII) oocytes. However, ATRX is undetectable in the chromosomes of meiotic spermatocytes at metaphase I and at the metaphase-anaphase transition. Metaphase I spermatocytes can be easily recognized by the association of SYCP2 protein (green) with centromeric domains. This also confirms that lack of ATRX staining in these chromosomes is not due to lack of antibody accessibility. Scale bar = 10 μm.

We have previously shown that the ATRX protein is present at centromeric heterochromatin domains in the chromosomes of metaphase-I and metaphase-II stage mouse oocytes [40]. However, the patterns of ATRX nuclear localization in the male germ line are not known. Therefore, we set out to determine the nuclear compartmentalization of ATRX during spermatogenesis. ATRX is present at centromeric heterochromatin domains in somatic testicular cells as well as in > 90% of spermatogonial cells obtained from pre-pubertal mice (Figure 6A). However, ATRX was undetectable in pachytene stage spermatocytes exhibiting full synapsis of homologous chromosomes as determined by simultaneous staining of the axial/lateral elements of the synaptonemal complex with the meiosis-specific marker SYCP3 (green) Figure 6 (A–B). In contrast, ATRX remains associated with centromeric heterochromatin domains in pachytene stage oocytes (Figure 6B). During male meiosis, chromosome bivalents at the metaphase-I stage retain SYCP2 staining at centromeric domains [41,42], but lack ATRX staining (Figure 6B). In contrast, during female meiosis, metaphase chromosomes show prominent ATRX staining at pericentric heterochromatin (Figure 6B). ATRX remains undetectable in male chromosomes at the anaphase to metaphase-II transition however, prominent ATRX signals were found associated with the chromocenter in approximately 10% of round spermatids. The majority of elongated spermatid nuclei or differentiating sperm heads showed negligible ATRX protein expression (Figure 6A). Thus, in contrast with neonatal spermatogonia in which the ATRX protein marks the entire Y chromosome, meiotic spermatocyte nuclei lack ATRX staining. These results indicate that the epigenetic composition of the Y chromosome during spermatogenesis is highly dynamic and that the patterns of ATRX nuclear localization during meiosis show marked differences in the male versus the female germ line.

## Discussion

In the present study, we provide evidence for the localization of repressive histone and chromatin modifications such as H3K9_me3 _and ATRX and the lack of transcriptionally permissive histone modifications such as H3K4_me2 _at pericentric heterochromatin and the Y chromosome of mouse neonatal spermatogonia. To our knowledge, these results provide the first evidence indicating that chromosomal 5-methyl cytosine patterns and histone methylation patterns might be regulated by independent mechanisms in neonatal spermatogonia. Interestingly, chromatin immunoprecipitation studies using MEFs revealed a specific association of both ATRX and H3K9_me3 _with pericentric repetitive sequences on the Y chromosome in somatic cells suggesting that binding of ATRX to the Y chromosome is not restricted to the germ line. In spite of its extensive global DNA hypo-methylation, the Y chromosome occupies a transcriptionally silent nuclear domain during interphase suggesting that H3K9_me3 _and ATRX might be important to maintain global transcriptional repression on the heterochromatic Y chromosome. Furthermore, our results indicate that epigenetic modifications on the Y chromosome during mammalian spermatogenesis are highly dynamic as indicated by the lack of nuclear ATRX staining at the pachytene stage and the chromosomes of metaphase-I or metaphase-II stage spermatocytes.

### Independent Regulation of Global DNA methylation and Histone Methylation in the Chromosomes of Neonatal Mouse Spermatogonia

The mammalian germ-line exhibits a unique program for genome reprogramming and transcriptional regulation. Although the majority of studies have focused on the analysis of post-meiotic haploid gene expression and chromatin remodeling during spermiogenesis [43-45] elegant cytogenetic analyses revealed striking changes in global DNA methylation at centromeric heterochromatin domains in neonatal spermatogonia [10,12]. However, whether this unique distribution of chromosomal DNA methylation affects the patterns of histone modifications and/or the recruitment of pericentric heterochromatin binding proteins remained to be determined. Our results suggest that in spite of the lack of global DNA methylation both ATRX and H3K9_me3 _remained faithfully associated with pericentric heterochromatin domains in the chromosomes of mouse neonatal spermatogonia. Furthermore, lack of ATRX and H3K9_me3 _throughout the highly methylated chromatids of the same autosomes provides additional evidence for an independent regulation between global DNA methylation and histone methylation during early postnatal development of the male germ line.

Notably, these repressive chromatin modifications were also prominent in the unmethylated Y chromosome arms in both neonatal spermatogonia and MEFs. This is consistent with previous studies indicating that H3K9_me3 _marks the Y chromosome in embryonic stem cells and 3T3 mouse fibroblasts [46,47]. Our results however also revealed that presence of 5-mC at pericentric heterochromatin and the Y chromosome is not required for H3K9 methylation at least in its tri-methylated form. On the other hand, in addition to H3K9_me3 _and ATRX other factors might be required for the subsequent re-establishment of chromosomal 5-mC patterns at pericentric heterochromatin upon differentiation of neonatal spermatogonia.

The lack of ATRX association with Y chromosome-specific repeat sequences observed in peripheral lymphocytes both on a chromosome-wide as well as at the molecular level, might constitute yet another example of the unique epigenetic status of the lymphocyte genome. In support of this idea, the global DNA methylation of the human [11] and mouse Y chromosome in peripheral lymphocytes remains high compared with MEFs and neonatal spermatogonia (see Additional file 1). Moreover, accumulating evidence indicates that the epigenetic composition of heterochromatic domains in mouse lymphocytes might be dramatically different from other somatic cell types. For instance, in female mouse lymphocytes, the inactive X chromosome lacks macroH2A association, an otherwise common marker of the late replicating inactive X chromosome [48]. Moreover, expression of some common heterochromatin-associated factors such as HP1 and macroH2A was shown to be dramatically reduced upon terminal differentiation of human leukocytes [49].

The complex interactions between DNA methylation and H3K9_me3 _in mammals are only beginning to be unraveled. For example, in somatic cells, DNA methylation seems to be necessary and sufficient for the establishment of some histone modifications, including di-methylation of H3K9 [50,51]. Consistent with this hypothesis, disruption of DNA methylation in LSH deficient mice results in the abnormal localization of H3K4_me2 _to pericentric heterochromatin domains in fibroblast cells [52]. However, the patterns of chromosomal 5-mC staining as well as H3K4_me2 _in neonatal spermatogonia from LSH deficient mice were indistinguishable from those of heterozygous controls. Thus, our results suggest that in contrast to somatic cells LSH might not be directly involved in regulating changes in di-methylation of H3K4 or in the establishment of chromosomal 5-mC patterns in the germ line. The potential relationship between DNA methylation at the single nucleotide level (CpG methylation) and the global DNA methylation patterns established by the localization of 5-mC throughout an entire chromosomal domain such as pericentric heterochromatin is not fully understood. However, CpG methylation at promoter regions of single-copy genes has been shown to co-exist with lack of chromosomal 5-mC staining on the human inactive X-chromosome [53]. Both major and minor satellite sequences as well as retrotransposons of the intracisternal A particle (IAP) class are protected from undergoing complete demethylation during genomic reprogramming in prenatal gonocytes in order to prevent a deleterious reactivation of retroviral elements [2-4,54]. Thus, CpG methylation at tandem repeats might be maintained by a strategy independent of the mechanism(s) regulating the establishment of chromosomal 5-mC patterns in neonatal spermatogonia.

DNA methylation patterns in the mammalian germ line are established by a family of DNA cytosine-5 methyltransferases (DNMTs) [55,56]. DNMT3a and DNMT3L are present in prenatal gonocytes and translocate into the nucleus on day 17.5 p.c. during the initial establishment of *de novo *DNA methylation patterns [38,57-59]. Importantly, DNMT3a and DNMT3L are directly involved in the establishment of DNA methylation for imprinted genes as well as transcriptional repression of retrotransposons in the male germ line [60-62]. However, the methylation of tandem repeats at centromeric heterochromatin in DNMT3L deficient male mice was not affected [60]. It is conceivable that in the absence of 5-mC at pericentric heterochromatin in neonatal spermatogonia H3K9_me3 _and ATRX might play a role in transient repression of repetitive elements in order to prevent their transcriptional reactivation in the spermatogonial cell genome. Thus, histone methylation and recruitment of heterochromatin binding proteins may be essential for maintaining the transcriptional quiescence of pericentric heterochromatin in neonatal spermatogonia before the patterns of 5-methyl cytosine are re-established to these chromosomal domains upon subsequent differentiation of proliferating spermatogonia in order to provide a more stable repression mark.

### Pre-meiotic heterochromatinization and global transcriptional silencing of the Y chromosome

Previous studies suggested that in several mammalian cell lines DNA on both sex chromosomes is hypo-methylated compared with the rest of the autosomes [11] and that the morphologically condensed and heterochromatic Y chromosome is hypo-methylated in neonatal spermatogonia [12]. Use of X and Y-chromosome specific probes after 5-methyl cytosine staining allowed us to confirm the hypo-methylation status of the Y chromosome and unequivocally establish that the second hypo-methylated chromosome observed in neonatal spermatogonia corresponds to the X chromosome. In adult type A and type B spermatogonia the Y chromosome lays within a transcriptionally quiescent nuclear domain, although it is well established that some Y-linked genes involved in spermatogenesis are expressed within this heterochromatic environment [37,63]. Our results extend these observations to the analysis of neonatal spermatogonia and indicate that in spite of the lack of 5-mC staining, global transcriptional repression of the Y chromosome is established prior to differentiation of spermatogonial cells and well in advance from the process of meiotic sex chromosome inactivation. Evidence obtained in several species ranging from flies to mammals suggests that the Y chromosome consists of about 95% heterochromatin. Heterochromatinization and the erosion of transcriptional activity on the Y chromosome might be a consequence of the accumulation of large numbers of retroelements and the requirement to silence these foreign sequences [64]. Thus, in the absence of global DNA methylation, repressive histone modifications such as H3K9_me3 _and ATRX as well as the lack of H3K4_me2 _associated with repetitive sequences might play an important role in silencing deleterious repetitive elements on the Y chromosome during the critical perinatal period for transposon silencing in the male germ line [60].

### Dynamic epigenetic composition of the Y chromosome during spermatogenesis

Sequential changes in histone modifications on the sex chromosomes have been observed in mouse spermatocytes from the pachytene stage onwards [65,66]. Importantly, several of these modifications, which are known to be associated with transcriptional inactivation, were recently shown to persist throughout spermiogenesis [37,67]. Both sex chromosomes were found enriched for H3K4_me2 _in adult spermatocytes and round spermatids at a time when sex chromatin is transcriptionally silent [65]. In contrast, our results indicate that the Y chromosome in neonatal spermatogonia lacks H3K4_me2 _suggesting that the epigenetic composition of the Y chromosome during spermatogenesis is highly dynamic. In the female germ line, the ATRX protein binds to pericentric heterochromatin at the pachytene stage (this study) as well as in oocytes at the metaphase I and metaphase II stage [40]. However, localization of the ATRX protein with centromeric heterochromatin during spermatogenesis was limited to pre-meiotic spermatogonial cells and a subset of round spermatids with rather large nuclei and one or two prominent chromocenters. Interestingly, DNA methylation at juxtacentromeric regions is heterogenous and remains low in pachytene stage oocytes and during the second meiotic division. In contrast, centromeric heterochromatin was found to be prominently methylated during meiotic progression in male germ cells, indicating that sex-specific differences in global 5-mC levels exist [68]. Differences in the patterns of ATRX nuclear localization during spermatogenesis versus oogenesis as well as the absence of ATRX staining on the Y chromosome in mouse lymphocytes suggest that the epigenetic composition of the Y chromosome may be affected by cell type or stage of differentiation.

## Conclusion

**Figure 7:**
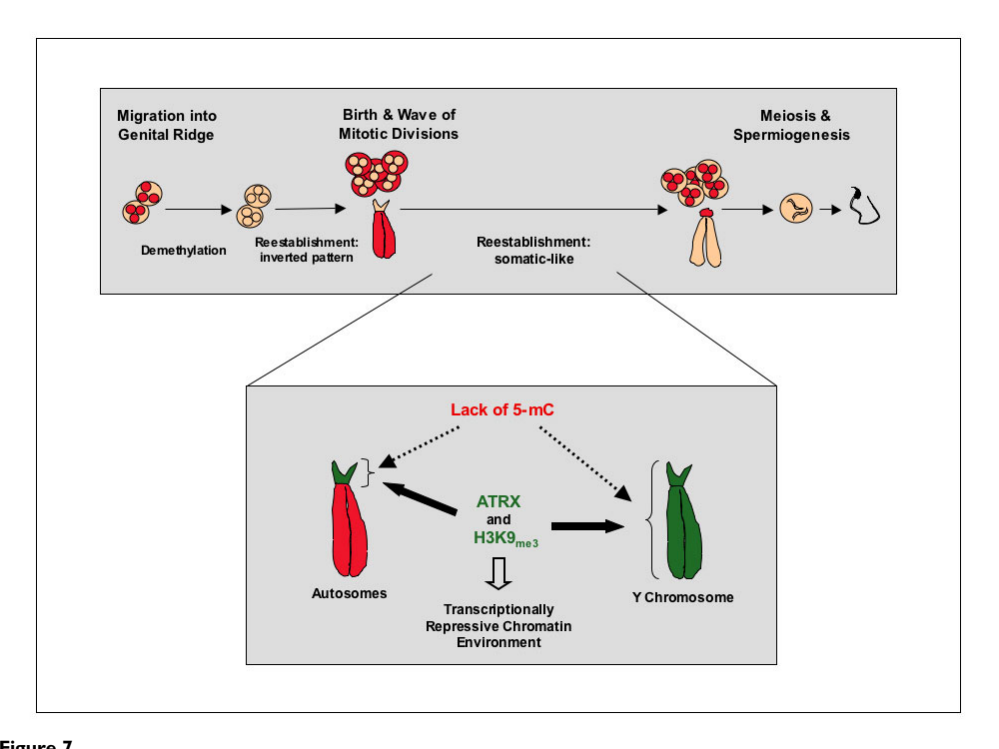
**Model for the establishment of a transcriptionally repressive chromatin environment at centromeric heterochromatin and the Y chromosome in neonatal spermatogonia**. The levels of CpG methylation previously observed at major and minor satellite sequences following genome reprogramming in fetal germ cells [2–4] are maintained within a centromeric heterochromatin environment lacking global methylation as determined by 5-mC staining [10]. Establishment of repressive histone and chromatin modifications such as H3K9_me3 _and ATRX are essential for the maintenance of a repressive chromatin configuration and might contribute to the mechanisms responsible for maintaining the transcriptional quiescence of potentially deleterious repetitive elements at tandem repeats. Histone methylation (H3K9_me3_) and association with chromatin remodeling proteins such as ATRX may precede the establishment of chromosomal 5-mC patterns as a mechanism to maintain a repressive chromatin environment at constitutive heterochromatin domains in neonatal spermatogonia. Although pericentric heterochromatin and the Y chromosome share similar chromatin marks, differences might exist on the mechanisms imposing these chromatin modifications as the Y chromosome lacks chromosomal 5-mC in both germ cells and somatic cells.

Maintenance of CpG methylation at major and minor satellite sequences following genome reprogramming in the germ line is essential for silencing the expression of potentially deleterious repetitive elements [2-4]. However, the mechanisms involved in this process are not known. Here we provide the initial evidence for the presence of repressive histone and chromatin modifications at pericentric heterochromatin domains in neonatal spermatogonia. We propose a model in which H3K9_me3 _and ATRX might contribute to the maintenance of a transcriptionally repressive chromatin environment in the absence of global DNA methylation (Figure 7). In this model, lack of chromosomal 5-mC at pericentric heterochromatin results from the global demethylation events taking place during genome reprogramming in fetal germ cells. Importantly, the establishment and/or maintenance of repressive histone and chromatin modifications may be essential for transcriptional silencing and for maintaining the levels of CpG methylation previously observed at tandem repeats following genome reprogramming [2-4]. The subsequent cytosine methylation observed at pericentric heterochromatin upon spermatogonial cell differentiation may thus reinforce a repressive chromatin structure initially nucleated by chromatin remodeling proteins and histone methylation. These results might have important implications for our understanding of the interaction between epigenetic control of gene expression and the potential for self-renewal in spermatogonial cells [69,70]. Moreover, further studies will be required to determine whether the association of ATRX with the mammalian Y chromosome has a functional implication for the onset of gonadal dysgenesis and the abnormal male sexual differentiation phenotypes observed in human patients with ATRX syndrome [71].

## Methods

### Chromosome analysis in neonatal spermatogonia and adult spermatocytes

All experiments were conducted in accordance with the guidelines of the Institutional Animal Care and Use Committees of the University of Pennsylvania. Testicular cells were obtained from neonatal (day 2 of postnatal development) and adult (> day 80) C57BL6/6J × SJL/J F1 mice. The day of delivery was designated as day 0. Briefly, the seminiferous tubules were digested with 1 mg/ml Collagenase (Sigma, St Louis, MO, USA) in PBS for 5 minutes at 37°C and dissociated by gentle pipetting. The cell suspension was then centrifuged at 1000 rpm for 5 minutes, washed in Minimum Essential Medium (MEM; GIBCO Life Technologies, Grand Island NY, USA) supplemented with 3 mg/ml Bovine Serum Albumin (BSA; Sigma). Surface spreads of spermatogenic cells were prepared immediately after enzymatic digestion of testes obtained from pre-pubertal and adult male mice as previously described [72]. Spermatogonial cells were also collected from homozygous (-/-) and heterozygous knockout male mice deficient for the LSH protein [28,73]. Mice homozygous for a null mutation on the LSH protein die within hours after birth [28]. Thus spermatogonial cells were obtained from fetuses at embryonic day 18.5 (E18.5) and cultured in MEM/BSA at 37°C under an atmosphere of 5% O_2_, 5% CO_2 _and 90% N_2 _for three to four days. Loosely attached spermatogonial cells were released from the monolayer of somatic cells that had formed during the culture period and transferred to fresh medium. The cell suspension containing segregated spermatogonia was then treated with 100 ng/ml Colchicine (GIBCO) and cultured for an additional 6–8 h. Metaphase spreads were prepared after hypotonic treatment in 75 mM KCl before final fixation with a solution of methanol/acetic acid (3:1). Chromosome spreads from adult wild type spermatogenic cells were also obtained by the method of squash preparation using isolated seminiferous tubules as described [74].

The analysis of histone methylation in chromosomes was conducted using antibodies specific for histone H3 tri-methylated on lysine 9 (H3K9_me3_; Abcam) and histone H3 di-methylated at lysine 4 (H3K4_me2_; Upstate) at a 1:200 and 1:2000 dilution in PBS, respectively. Analysis of global DNA methylation patterns was conducted after chromosome denaturation with 2N HCl for 20 minutes followed by extensive washing in PBS and using a mouse monoclonal antibody against 5-methylcytosine (5-mC; Calbiochem) at a 1:200 dilution. The anti-guinea pig antibody against synaptonemal complex protein 3 (SYCP3) was used at a 1:250 dilution [75]. The mouse monoclonal antibody against the chromatin remodeling protein ATRX [30] was used at a 1:5 dilution whereas the rabbit anti-ATRX antibody (Santa Cruz Biotechnology) was used at a 1:400 dilution. Primary antibodies were detected with Alexa Fluor^R^-conjugated secondary antibodies (Molecular Probes, Eugene, Oregon, USA) applied for 2 h at room temperature in a humidified chamber. Hoechst 33258 DNA stain was used to visualize condensed heterochromatin areas and cover slips were mounted with Vecta Shield antifading medium (Vector Laboratories, Inc. Burlingame, CA). Chromosome analysis was conducted using a Leica DMRE fluorescence microscope and images were captured using a Leica DFC 350F CCD camera.

### Fluorescence in situ hybridization (FISH)

Following immunochemistry, slides were processed for FISH analysis using a Cy3-conjugated Y-chromosome paint probe or, alternatively a fluorescein isothiocyanate (FITC) conjugated X- chromosome probe (Cambio Ltd., Cambridge, England), according to the manufacturer's specifications and with the following modifications. Briefly, surface spread spermatogonial nuclei were denatured in 70% formamide in 2× SSC at 80°C for 10 minutes and subsequently chilled on ice-cold 70% ethanol for 5 minutes. The X and Y chromosome probes were denatured for 7 minutes at 75°C and incubated at 41°C for 1 h. Overnight hybridization was carried out in a humidified chamber at 41°C. Stringency washes were conducted in a solution containing 50% formamide in 2× SSC as previously described [76].

### Chromatin Immunoprecipitation (ChIP)

Male mouse primary embryonic fibroblasts (MEFs) were isolated at E14.5 of fetal development and cultured for 3 passages before harvesting. Heparinized whole blood was cultured in PB-MAX medium (Invitrogen, Carlsbad, CA) for 72 h to obtain actively dividing peripheral lymphocytes. Chromatin immunoprecipitation was performed using the ChIP-IT™ kit (Active Motif; Carlsbad, CA). Briefly, cells were fixed for 10 minutes with 1% formaldehyde and chromatin fragments prepared by enzymatic shearing following manufacturer's instructions. Experiments were carried out in triplicates using an antibody for the transcription factor TFIIB (Santa Cruz) as a positive control, as well as antibodies for the ATRX protein (Santa Cruz), anti-H3K4_me2 _(Upstate) and an anti-H3K9_me3 _(Abcam) antibody. A pre-immune IgG (Active Motif) was used as a negative control for the immunoprecipitation step. Precipitated chromatin was amplified by PCR with primers corresponding to clone pEMS666 containing pericentric repetitive sequences on the Y chromosome with forward sequence 5'-GCTAGGCTTGGGTTTTGTTG-3' and reverse primer 5'-GCAGTAAGTAGGTGGAGAGA-3' [77]. Conditions for PCR amplification were as follows, denaturation 94°C 3 min, followed by 36 cycles of denaturation at 94°C for 45 seconds, annealing 58°C 45 seconds, elongation 72°C 45 seconds and a final elongation step for 5 minutes.

### Transcription run-on assays

To determine subnuclear domain-specific transcriptional activity in neonatal spermatogonia, nascent transcripts were detected after 5-bromouridine 5'triphosphate (Br-UTP; Sigma), incorporation as described previously [78]. Following a brief permeation of the plasma membrane with 0.03% Triton X (Biorad) in PBS for 2 minutes, the spermatogonial cell suspension was rinsed in fresh PBS and then transferred to transcription buffer supplemented as described [78] for 40 minutes at 37°C before fixation in 4% Paraformaldehyde (PFA; EMS Hatfield, PA) supplemented with 0.15% Triton X. Transcriptional activity was detected with an anti-bromodeoxyuridine (BrdU, Boehringer Mannheim) antibody used at a concentration of 2 μg/ml followed by detection with an Alexa Fluor-488 secondary antibody (Molecular Probes). The nuclear domain corresponding to the Y chromosome in transcriptionally active cells was visualized by FISH while germ cells and somatic cells were subsequently distinguished by their corresponding 5-mC staining patterns as described above.

### Statistical Analysis

Individual band intensity after chromatin immunoprecipitation was quantified using the Kodak 1D Image Analysis Software (Kodak, Rochester, NY). Data are presented as the mean net intensity (× 10^-3^) of at least three independent experiments. Statistical analysis was performed by one-way analysis of variance (ANOVA) and comparison of all pairs by Tukey-Kramer HSD using JMP Start Statistics (SAS Institute Inc., Cary, NC). Variation among replicates is presented as the standard error of the mean (SEM). Differences were considered significant when (*P *< 0.05).

## Authors' contributions

CB designed and conducted the experiments, data analysis, and participated in drafting and editing the manuscript. AS was involved in the analysis of LSH samples. KM generated LSH knockout mice and contributed to the writing of the manuscript. RDLF designed and supervised the study and wrote the manuscript. All authors read and approved the final manuscript.

## Supplementary Material

Additional file 1 **Comparison of global DNA methylation patterns in the Y chromosome of mouse peripheral lymphocytes and neonatal spermatogonia**. **A**) In peripheral lymphocytes, the Y chromosome (green) remains methylated as determined by 5-mC staining (red; inset and bold arrow) and is thus indistinguishable from the rest of the autosomes. In contrast the Y chromosome of primary fibroblasts (**B**) and neonatal spermatogonia (**C**) is subject to an extensive demethylation that renders it easily discernible from the rest of the autosomes (compare inset in **A **with insets in **B-C**). Thin arrows point to centromeric heterochromatin, while bold arrows mark the location of the Y chromosome. The intense 5-mC staining on chromatids of peripheral lymphocytes also precludes the clear distinction between chromatids and centromeric heterochromatin in comparison to primary fibroblasts (thin arrow; **A**). Click here for file
