# Supplementary figures and images for: Association of ATRX with pericentric heterochromatin and the Y chromosome of neonatal mouse spermatogonia
Source: BMC Mol Biol. 2008 Mar 13;9:29. doi: 10.1186/1471-2199-9-29 (PMC2275742; doi:10.1186/1471-2199-9-29)

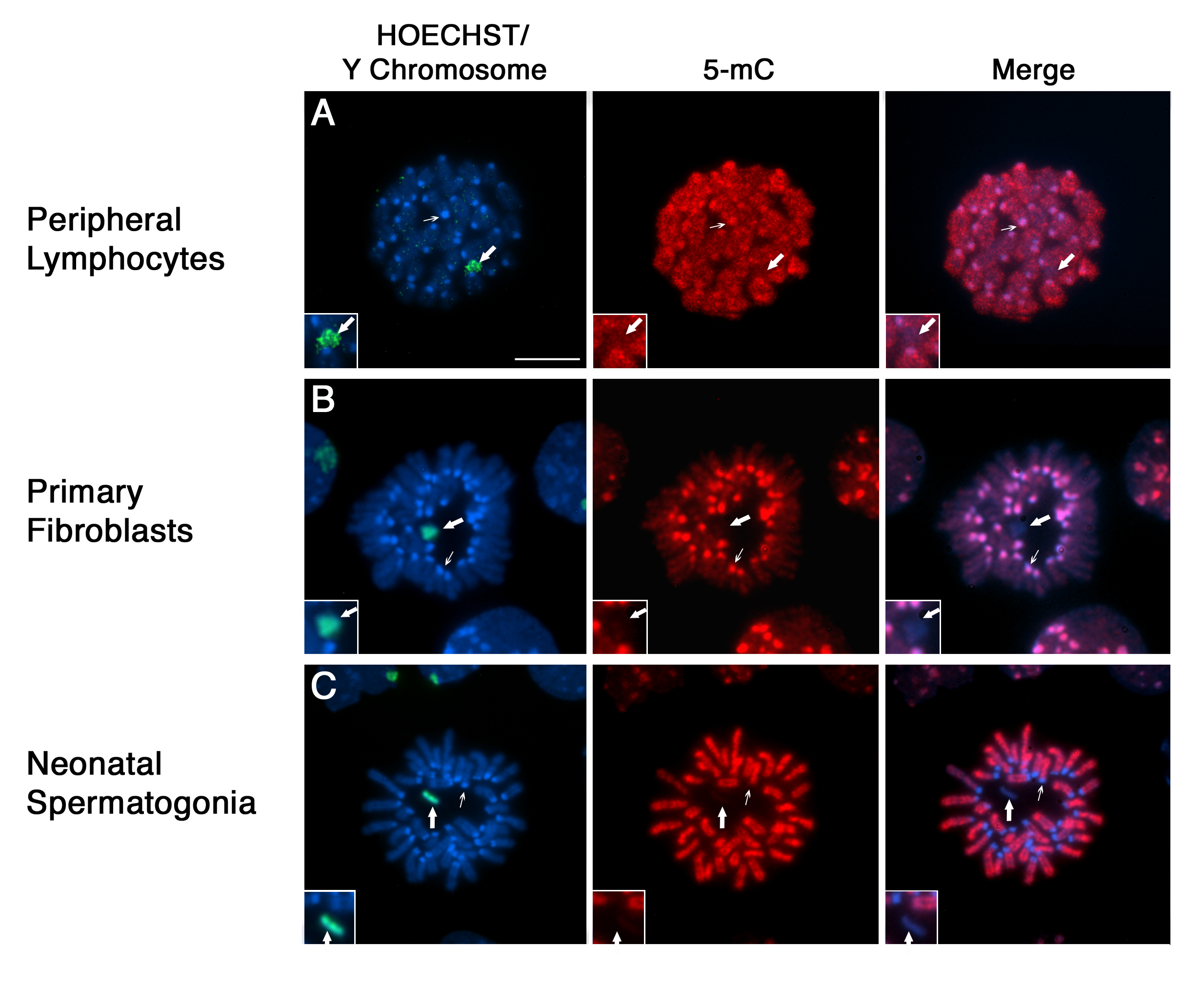

Supplement: Additional file 1 — Comparison of global DNA methylation patterns in the Y chromosome of mouse peripheral lymphocytes and neonatal spermatogonia. A) In peripheral lymphocytes, the Y chromosome (green) remains methylated as determined by 5-mC staining (red; inset and bold arrow) and is thus indistinguishable from the rest of the autosomes. In contrast the Y chromosome of primary fibroblasts (B) and neonatal spermatogonia (C) is subject to an extensive demethylation that renders it easily discernible from the rest of the autosomes (compare inset in A with insets in B-C). Thin arrows point to centromeric heterochromatin, while bold arrows mark the location of the Y chromosome. The intense 5-mC staining on chromatids of peripheral lymphocytes also precludes the clear distinction between chromatids and centromeric heterochromatin in comparison to primary fibroblasts (thin arrow; A). [file 1471-2199-9-29-S1.jpeg]
